# Rehabilitation Treatment for Shoulder Pain in Parkinson’s Disease: A Pilot Study

**DOI:** 10.3390/jcm14041127

**Published:** 2025-02-10

**Authors:** Emanuele Amadio, Luca Cimini, Ilaria Ruotolo, Alessandra Carlizza, Anna Berardi, Andrea Marini Padovani, Giovanni Sellitto, Giovanni Fabbrini, Giovanni Galeoto

**Affiliations:** 1 Department of Human Neurosciences, Sapienza University of Rome, 00185 Rome, Italy; ilaria.ruotolo@uniroma1.it (I.R.); anna.berardi@uniroma1.it (A.B.); andreamarinip@gmail.com (A.M.P.); giovanni.sellitto@uniroma1.it (G.S.); giovanni.fabbrini@uniroma1.it (G.F.); giovanni.galeoto@uniroma1.it (G.G.); 2 Department of Human Neurosciences, School of Physiotherapy, Sapienza University of Rome, 00185 Rome, Italy; luca.cimini11@gmail.com; 3 Department of Public Health and Infectious Diseases, Sapienza University of Rome, 00185 Rome, Italy; 4 Faculty of Medicine, UniCamillus, International Medical University in Rome, Via di Sant’Alessandro, 8, 00131 Rome, Italy; alessandra.carlizza@unicamillus.org; 5 IRCSS Neuromed, Via Atinense, 18, 86077 Pozzilli, Italy

**Keywords:** mobility, musculoskeletal, outcome, pain, physiotherapy

## Abstract

**Background/Objectives:** Due to rigidity, musculoskeletal pain is more common in people with Parkinson’s disease (PD) compared with age-matched older adults, and the shoulder is one of the body parts that is most involved. In the literature, there is no clear standard for the treatment of shoulder pain in people with PD. This clinical trial study aimed to evaluate the effectiveness of physiotherapy treatment for people with PD with painful shoulders. **Methods:** The main goals were improvements in pain intensity, balance, quality of life (QoL), and activities of daily living (ADL), evaluated with the Parkinson’s Disease Questionnaire 39 (PDQ-39), Berg Balance Scale (BBS), Community Integration Questionnaire (CIQ-R), 12-Item Short-Form Survey (SF-12), Disabilities of the Arm, Shoulder, and Hand (DASH) scale, and Numeric Pain Rating Scale (NPRS). Also, the evaluation comprised range of motion (ROM) evaluation with a goniometer and the Medical Research Council (MRC) scale. The inclusion criteria of this study were a diagnosis of PD associated with shoulder pain, and a stage of disease of 1–2 on the Hoehn and Yahr scale. **Results:** The sample comprised 16 participants; the mean age of the participants was 72. Through feedback collected from the individuals participating in this study, it emerged that the rehabilitation approach specifically designed for individuals suffering from shoulder pain associated with Parkinson’s disease produced remarkable results. **Conclusions:** These results were confirmed by a series of statistically significant data, which showed significant improvements in several areas: joint mobility, muscle strength, motor coordination, the ability to perform daily activities, emotional state, pain reduction, QoL improvement, and balance in both dynamic and static conditions.

## 1. Introduction

Parkinson’s disease (PD) affects 1–2 individuals per 1000 of the population at any time. PD prevalence is increasing with age, and PD affects 1% of the population aged above 60 years. It is a multifaceted neurodegenerative disorder characterized by both motor and non-motor symptoms. Motor symptoms, including bradykinesia, rigidity, rest tremor, and postural instability, are classically associated with dopaminergic neuronal loss in the substantia nigra [1,2]. Non-motor symptoms, such as autonomic dysfunction, cognitive decline, psychiatric disturbances, and sleep disorders, often precede motor symptoms by many years, and significantly impact the patient’s quality of life [2]. Dysautonomia, a common non-motor feature, includes gastrointestinal dysfunction, urinary disturbances, and other autonomic failures. Not only is it a key clinical feature of PD, but it is also crucial in differentiating PD from atypical parkinsonism, like multiple-system atrophy (MSA) and progressive supranuclear palsy (PSP), where the presentation and extent of autonomic involvement vary. For instance, MSA shows severe autonomic dysfunction, including prominent urinary incontinence, while PSP typically presents milder autonomic symptoms, often associated with cognitive decline [1]. Rigidity may be associated with pain, and a painful shoulder is one of the most frequent initial manifestations of PD; however, it is commonly misdiagnosed as arthritis, bursitis, or rotator cuff injury [3,4]. In addition, rigidity of the neck and trunk (axial rigidity) may occur, resulting in abnormal axial postures (e.g., anterocollis, scoliosis). Postural deformities, resulting in flexed neck and trunk posture and flexed elbows and knees, are often associated with rigidity. Other skeletal abnormalities include extreme neck flexion (“dropped head” or “bent spine”), truncal flexion (camptocormia), and scoliosis. Camptocormia is characterized by extreme flexion of the thoracolumbar spine. Another truncal deformity is Pisa syndrome, characterized by a tilting of the trunk, particularly when sitting or standing [5].

Musculoskeletal (MSK) pain is more common in people with PD compared with age-matched older adults [6]. The shoulder is one of the body parts that is most involved; the clinical characteristics of shoulder dysfunction in PD are as follows: (1) restricted movements due to shoulder rigidity, frozen shoulder, akinesia, arm swing loss, or muscle weakness; and/or (2) altered quality of shoulder motion due to arm tremor, bradykinesia, or pain. Shoulder pain and stiffness are the most frequent symptoms of shoulder dysfunction in PD. A retrospective analysis of 309 consecutive patients diagnosed with PD in our clinic revealed that 35 (11%) complained of shoulder pain, in some cases preceding the onset of motor symptoms by several years. The mechanism of shoulder pain in PD is unclear, although rigidity and bradykinesia, leading to immobility and subsequent shoulder dysfunction and discomfort, are possible explanations [7].

Riley and colleagues were the first to find a significantly higher incidence of a history of shoulder symptoms (43% vs. 23%) in 150 PD patients compared to 60 matched healthy controls. Kim and colleagues studied 400 PD patients and 138 age- and sex-matched controls, and found that the shoulder was more affected in the PD group than in the control group (15.0% vs. 8.7%) [8]. In the past year, 31% of the general elderly population reported experiencing shoulder pain for at least three consecutive months. When directly comparing people with PD and age-matched controls, shoulder pain or disturbance is more common in people with PD. When assessing their shoulder pain intensity at its worst, people with PD rated it as moderate to severe. There is no clear standard for pharmacologic, surgical, or rehabilitative treatment of shoulder pain and/or pathology in people with PD. Madden and Hall reported that 40% of people with PD with shoulder pain had improvement with PD-specific treatment (e.g., dopaminergic medication, deep brain stimulation). However, 40% of people with PD with shoulder pain reported that PD-specific treatment was not adequate for their shoulder complaints. To our knowledge, no studies have reported on the effects of rehabilitation on shoulder pain in people with PD [6]. This clinical trial study aimed to evaluate the effectiveness of physiotherapy treatment for people with PD with painful shoulders; improvements in pain intensity, balance, quality of life (QoL), and activities of daily living (ADL) were evaluated.

## 2. Materials and Methods

This pilot study was conducted by R.E.S. (Riabilitazione Evidenze e Sviluppo) research group from the Sapienza University of Rome (Italy), a research team with extensive expertise and significant involvement in numerous studies focusing on rehabilitation science and evidence-based therapeutic approaches [9,10,11,12,13].

Patients were recruited at the Department of Human Neurosciences “Policlinico Umberto I” in Rome in November 2022; all eligible participants were informed about the methods and aim of the study, and those interested provided their formal consent to participate in the rehabilitation protocols, in accordance with the Declaration of Helsinki. The inclusion criteria of this study were a diagnosis of PD, based on the clinical diagnostic criteria of the Movement Disorder Society for PD [14]; shoulder pain; a stage of disease, according to H&Y scale, of 1–2 [15]; and no concomitant pathologies before the diagnosis of PD. The sample consisted of 16 participants: 10 male and 6 female subjects.

### 2.1. Intervention

Physiotherapy intervention programs are usually based on 50–60 min treatment sessions that occur twice a week until 10 sessions have been undertaken. The intervention was focused on reducing pain, improving active and passive shoulder joint mobility, improving the perception of the limb in space, improving the quality of movement, improving muscle strength, returning to possible functional activities, and improving balance and gait cycle. In this study, the outcomes were subdivided into different groups: the primary outcomes were defined as a decrease in pain during shoulder movement and an improvement in the quality of movement of the whole shoulder complex, with work also carried out on the scapula and tension of the cervical tract muscles; the secondary outcomes included the restoring of a sufficient range of motion (ROM), the perception of shoulder and scapular movement in the space, muscle strength, and the execution of movements, in order to bring patients back to performing some ADL; the tertiary outcomes were related to the improvement of balance, trunk stability, and gait, in relation to the pendular movement of the upper limbs, in order to improve or restore a good gait cycle.

### 2.2. Assessment Tools

The tests administered in this study included five tools: the Berg Balance Scale (BBS) [16,17], the Disability of the Arm, Shoulder, and Hand (DASH) scale [18], the Parkinson’s Disease Questionnaire 39 (PDQ-39) [9,19], the 12-Item Short-Form Survey (SF-12) [20,21], and the Community Integration Questionnaire—Revised (CIQ-R) [22].

The BBS is a 14-item tool to assess balance ability. It examines the subject’s ability to maintain positions or movements of increasing difficulty, by diminishing the base of support from sitting and standing to a single-leg stance.

The Disabilities of the Arm, Shoulder, and Hand (DASH) scale is designed to be a complete instrument, since it assesses the upper limbs as one, and is not limited to a single body segment. The development of the DASH scale was based on three theoretical aspects (physical function, symptoms, and social function).

PDQ-39, developed by Peto, was used to evaluate the change in QoL of the patient between the start of physiotherapy and the end of treatment. This scale consists of 39 items, with five answers for each question, where the worst is the fifth answer, and the best is the first; the possible answers are “never”, “occasionally”, “sometimes”, “often”, and “always”. The scale is mainly subdivided into eight subscales: mobility (10 items), ADL (6 items), emotional well-being (6 items), stigma (4 items), social support (3 items), cognitive faculties (4 items), communications (3 items), and bodily discomfort (3 items).

The SF-12 scale is a type of scale that presents 12 items that measure 8 health domains. The items are subdivided into physical functioning (2 items), role limitations due to physical health problems (2 items), bodily pain (1 item), general health status (1 item), vitality (1 item), social functioning (1 item), role limitations due to emotional problems (2 items), and mental health status (2 items).

The CIQ-R is the most used outcome tool for measuring the participation and community integration of people with disabilities; it allows us to obtain an objective overview of community integration and participation in ADL and instrumental activities of daily living (IALD). This is a reliable and valid instrument to measure “home, social, productivity integration, and electronic social networking” in people with severe and disabling pathologies.

The Medical Research Council (MRC) scale [23] proposed uses the numeral grades 0–5. For grades 0–2, which indicate that movement against resistance is not possible, the forearm was held in a neutral position between pronation and supination and the wrist in a 0° position, with which the examiner assisted. Grade 3 of the MRC scale indicates that active movement against gravity is possible; grade 4 denotes active movement against resistance. The same resistance as that on the contralateral side is rated grade 5.

The Numeric Pain Rating Scale (NPRS) is a unidimensional measure of pain intensity in adults; it is a segmented numeric version of the visual analog scale (VAS), which prompts a respondent to select a whole number (0–10 integers) that best reflects the intensity of his/her pain [24].

### 2.3. Rehabilitation Protocol

The therapeutic intervention consisted of 10 physiotherapy sessions, carried out bi-weekly for 50–60 min each. The short-term goals focused on optimizing ADL and patients’ QoL, aiming to restore the maximum degree of post-pain reduction autonomy and improved shoulder mobility. Before the start of the physiotherapy treatment, a thorough functional assessment of the patients was carried out, from a general assessment to a specific assessment of the shoulder [25]. This assessment included data collection through a detailed history and administration of six different scales of evaluation (BBS, DASH, SF-12, PDQ-39, CIQ-R, NRS). Objective clinical examination of the shoulder started with observation, inspection, and palpation to assess the sites of pain and muscular stiffness. Functional evaluation allowed the development of a targeted treatment by identifying triggers. Postural analysis detected any compensations, particularly regarding the position of the humeral head. Measuring the range of motion (ROM), passive range of motion (PROM), and active range of motion (AROM) helped to provide an understanding of the direction of pain and impairment of the shoulder joint. Scapular mobility, perception, and movement control were analyzed by observation of active and passive movement. The analysis focused on the synergy of the shoulder and on how to ensure complete mobility. The muscle test, preceded by the evaluation of every single muscle, followed the protocol of the book *Musculoskeletal Assessment* [26] to assess muscle strength in the muscles of the upper limb that make up the shoulder complex. These muscles were analyzed using the MRC [23]. Joint stability tests were essential to detect hypermobility or joint laxity, evaluating the contribution of ligaments, tendons, and muscles to shoulder support. After the evaluation, a treatment based on the analysis of the data collected through functional examinations and the use of assessment scales was customized, aiming at providing the most appropriate treatment for each patient’s specific conditions. The first treatment phase was dedicated to analyzing the joint complex, evaluating painful structures and the patient’s reaction to passive and active mobilization, and paying particular attention to pain. The objective was gradual, aimed at the restoration of functional conditions, considering individual variations in deficits. Progressive restoration of the full range of shoulder movement is crucial to improve QoL and the ability to perform daily activities when shoulder use is limited. During the analysis, a non-physiological position of the humeral head was often found, associated with abnormal posture such as camptocormia. Manual mobilization addressed this issue by assessing humerus mobility, capsule stiffness, and muscle tension. The next focus was gradually repositioning the humeral head, using passive mobilization in external rotation to correct the wrong muscle recruitment and restore the complete range of movement. In addition, it was essential to address the common conditions of the rotatory muscles [27,28], by focusing specifically on the subhead muscle, the shortening of which is often responsible for the internal rotation and anteposition of the humeral head. The authors also worked on reducing the muscular tension of the small pectoralis, another contributor to the internal rotation and anteposition of the shoulder, and the anterior dentate muscle, which can cause shoulder adhesions and restrict proper mobility [29]. In addition, we addressed the need to treat muscles that can limit the correct movement of the shoulder and contribute to stiffness, including the brachial biceps, the large and small pectoralis, the serratus anterior muscle, the trapezium, the subscapularis, and all the neck muscles associated with cervical pain. This preparatory phase enabled these muscles, particularly those with high stiffness and shortening, to function optimally during the next active exercise phase. In the next stage of treatment, attention was paid to mobilization of the scapula in all directions, focusing on crucial movements over 60 degrees in the scapula–thoracic joint. This approach was crucial to ensure the proper functioning of the joint and promote effective muscle recruitment, especially in the last degrees of movement. However, it was a priority first to restore the proper position of the humeral head and check for proper mobility in the glenohumeral joint in the first degrees of movement. Once the patient had achieved a correct humeral head position and good shoulder mobility, muscle reinforcement was achieved using short-lived isometric contractions or active exercises without gravitational resistance. During this phase, we focused on training sensory perception and awareness of the upper limbs, using visual or tactile feedback to guide the patient in the correct execution of movements. This part was crucial in order to identify the muscles involved in active exercise and develop an accurate understanding of the movements involved in muscle strengthening and motor control. The goal was to achieve pain-free movement coordination in all components of the shoulder, achieving adequate body balance to maintain stability and safety in daily activities and walking. The exercises aimed to improve mobility, stretch and strengthen muscles, develop the perception of movement in the shoulder, arm, and hand, and improve the coordination of movements. The progression of the exercises was customized according to the patients’ individual stiffness levels and pathological conditions.

### 2.4. Data Analysis

There were sixteen subjects selected, and only one interrupted the cycle. The participants were divided into ten males and six females, with a mean age of 72 and a standard deviation of 9.4. All the participants included had a score of 1–2 on the Hoehn and Yahr (H&Y) scale. For inferential analysis, the Wilcoxon signed-rank test was used to calculate the statistical significance of the values obtained from the evaluation scales and tools. The program used for the inferential analysis was SPSS version 27. The significance level set for the *p*-value was less than or equal to 0.05 (*p* ≤ 0.05). The general hypothesis, made at the beginning of the treatment, was based on the expectations of possible functional improvements. When we used the BBS, DASH, PDQ-39, SF-12, and CIQ-R and performed muscle testing and goniometrical measurements on the affected shoulder, we found that the rehabilitative intervention had the role of reducing pain and improving range of motion (ROM), the strength and ability of the shoulder and hand, ADL, and QoL.

## 3. Results

Sixteen subjects, previously selected according to the defined inclusion criteria, were fully informed about the study’s purposes and modalities. The sample consisted of 16 participants, divided into 10 male and 6 female subjects. The mean age of the participants was 72, with a standard deviation of 9.4. All participants included in the study were between H&Y scale scores 1 and 2, indicating a specific stage of Parkinson’s disease progression.

The collected data were analyzed and presented in tables. These tables represent data collected before treatment (T0), after treatment (T1), 1 month after treatment (T2), and 3 months after treatment (T3).

Table 1 reports data from the assessment tools administered during treatment (T0 and T1), at the follow-up after 1 month (T2), and at the follow-up after 3 months (T3). From the evaluation through the Wilcoxon test, it can be asserted that we obtained a statistically significant improvement for the Berg Balance Scale at T0–T1, T2, and T3; a statistically significant improvement with the DASH scale at T0–T1, T2, and T3; and for PDQ-39, we obtained statistically significant data for all the items at T0–T1, T2, and T3, except for stigma, social support, and cognition. For the SF-12, the evaluation asserted a statistically significant improvement only for the PCS-12 at T0–T1. At last, for the NPRS, we obtained a statistically significant improvement only in the active left arm at T0–T1.

Table 2 reports data on ROM collected during treatment at T0 and T1, at the follow-up after 1 month at T2, and after 3 months at T3. From the evaluation through the Wilcoxon test, it can be asserted that we obtained a statistically significant improvement for the right arm in flexion and abduction at T0–T1 and T2, and in external rotation of the arm at the side at T0–T1, T2, and T3; for the active right arm, we obtained statistically significant results for flexion, abduction and external rotation of the arm at the side at T0–T1, T2, and T3, and also for extension only at T0–T1. For the passive left arm, a statistically significant improvement in flexion and abduction was reported only at T0–T1 and T2; we obtained a statistically significant improvement in T0–T1, T2, and T3 for the external rotation of the passive arm at the side. In conclusion, for the active ROM of the left arm, we reached statistically significant data for flexion, abduction, and external rotation of the arm at the side at T0–T1, T2, T3, and for extension at T0–T1.

Table 3 reports data from the MRC scale collected during treatment at T0 and T1, at the follow-up after 1 month at T2, and after 3 months at T3. From the evaluation through the Wilcoxon test, it can be asserted that we obtained a statistically significant improvement for all the muscles at T0–T1. The only muscles with statistically significant results at T2 were the deltoid, trapezium, triceps, biceps, pectoralis major, latissimus dorsi, and rhomboid muscles. At T3, statistically significant data were only reported for the deltoid and triceps. 

## 4. Discussion

This pilot study demonstrated significant improvements in several key outcomes for patients with Parkinson’s disease experiencing shoulder pain. Statistically significant enhancements were observed in range of motion (ROM), particularly in active bending, abduction, and external rotation, highlighting the effectiveness of the rehabilitation protocol. Pain reduction and improved functionality were reflected in DASH scores, while improvements in balance, as measured by the Berg Balance Scale (BBS), underscored the broader benefits of the intervention. Gains in quality of life (QoL), as evidenced by the PDQ-39 and CIQ-R scores, further emphasized the positive impact of the protocol on the participants’ ability to perform daily activities and integrate socially. These findings align with the broader understanding that targeted physiotherapy interventions can address not only physical limitations, but also functional and psychological outcomes, contributing to the overall well-being of patients with Parkinson’s disease. The study also demonstrated increased muscle strength across multiple muscle groups, which is a critical aspect for improving motor performance and reducing the functional impact of shoulder pain. While a modest decline in strength was observed during follow-ups at one and three months post-intervention, significant improvements in shoulder mobility, pain reduction, and functional capabilities were maintained. These retained benefits positively influenced daily activities and motor tasks, highlighting the long-term value of the intervention, despite the natural challenges associated with progressive neurological conditions such as Parkinson’s disease. The results of the study also revealed significant advancements in static and dynamic balance, as evidenced by the BBS scores. This improvement is particularly noteworthy given the direct relationship between balance and gait kinetics, which are often compromised in patients with Parkinson’s disease. By addressing shoulder pain and improving upper limb function, the intervention indirectly influenced postural control and gait stability. These findings align with previous literature that underscores the role of physiotherapy in enhancing motor and non-motor functions in Parkinson’s disease. Importantly, the benefits observed at the three-month follow-up validate the efficacy of the rehabilitation protocol in promoting sustained improvements in multiple domains, including ROM, pain reduction, and balance. From a broader perspective, this study highlights the importance of addressing shoulder pain as part of a comprehensive rehabilitation strategy for Parkinson’s disease. Musculoskeletal pain, particularly in the shoulder, is a frequently overlooked but highly impactful aspect of the disease that significantly affects patients’ quality of life and daily functionality. By incorporating targeted interventions for musculoskeletal pain into clinical practice, clinicians can improve not only physical outcomes, but also emotional well-being and social participation. The integration of personalized rehabilitation protocols, such as the one explored in this study, may offer a viable pathway for optimizing care for patients with Parkinson’s disease and associated shoulder pain. One of the main limitations of this study is the absence of a specific analysis of the clinical subtypes of Parkinson’s disease. This lack of differentiation may have influenced the observed outcomes, as variations in clinical manifestations and responses to rehabilitation treatments among subtypes could play a significant role. Another limitation of this study is the small sample size, which restricts the generalizability and robustness of the findings. This limitation was primarily due to the difficulty in recruiting patients with Parkinson’s disease in its early stages. Patients in the initial stages of Parkinson’s disease do not always report shoulder pain as a primary concern, which made it challenging to identify eligible participants for the study. Moreover, the absence of a control group, while allowing the study to evaluate the rehabilitation protocol’s effectiveness in a real-world clinical setting, limits the ability to draw definitive conclusions about the intervention’s efficacy compared to other treatments or natural disease progression. Future randomized controlled trials with larger sample sizes are essential to strengthen the evidence and provide clearer insights into the intervention’s benefits. Through feedback collected from individuals participating in this study, it emerged that the rehabilitation approach specifically designed for individuals suffering from shoulder pain associated with Parkinson’s disease produced remarkable results. These results are confirmed by a series of statistically significant data, which show significant improvements in several areas: joint mobility, muscle strength, motor coordination, the ability to perform daily activities, emotional state, pain reduction, QoL improvement, and balance in both dynamic and static conditions. Therefore, the proposed rehabilitation approach could prove highly beneficial in addressing shoulder pain and other issues related to Parkinson’s disease. This finding suggests that physiotherapists should carefully consider using this approach in the management of patients suffering from this pathology, and avoid underestimating the importance of treating shoulder pain in these clinical cases.

## 5. Conclusions

This pilot study demonstrated the effectiveness of a targeted rehabilitation protocol in improving shoulder pain, mobility, balance, and quality of life in patients with Parkinson’s disease. Significant enhancements in range of motion, muscle strength, and functional capabilities were observed, contributing positively to daily activities and social integration. Despite limitations such as a small sample size and the absence of a control group, the findings highlight the potential of personalized rehabilitation strategies to address musculoskeletal pain and its broader impact on Parkinson’s disease management. Future research should focus on larger, randomized trials to further validate these results and explore the integration of such protocols into standard clinical practice.

## Figures and Tables

**Table 1:** Wilcoxon test at T0 and T1 (5 weeks), T2 (9 weeks), and T3 (17 weeks) for Berg Balance Scale (BBS), Community Integration Questionnaire—Revised (CIQ-R), Numeric Pain Rating Scale (NPRS), Parkinson’s Disease Questionnaire 39 (PDQ-39), and 12-Item Short-Form Survey (SF-12)—Physical Component Summary (PCS) and Mental Component Summary (MCS).

	T0		T1		Wilcoxon T0–T1	*p*	T2		Wilcoxon T0–T2	*p*	T3		Wilcoxon T0–T3	*p*
	Mean ± sd	Median	Mean ± sd	Median			Media ± sd	Median			Media ± sd	Median		
BERG	46.40 ± 10.55	50	51.40 ± 8.67	55	−3.194	0.001 *	53.29 ± 1.38	53.00	−2.375	0.018 *	53.50 ± 1.52	53.50	−2.207	0.027 *
CIQ-R	17.28 ± 6.43	18	18.40 ± 5.99	19	−1.372	0.170	17.43 ± 6.19	16.00	−0.738	0.461	17.67 ± 6.74	18.00	−0.136	0.892
DASH	38.22 ± 23.95	33.30	20.94 ± 19.10	15	−3.409	0.001 *	24.99 ± 13.67	28.30	−2.371	0.018 *	29.87 ± 13.61	31.25	−2.207	0.027 *
PDQ-39														
Mobility	11.87 ± 10.32	12	8.73 ± 8.55	8	−2.324	0.020 *	8.29 ± 5.99	9.00	−1.687	0.092	9.83 ± 6.01	10.00	−1.802	0.072
ADL	8.27 ± 5.04	8	6.13 ± 4.66	5	−2.179	0.029 *	6.71 ± 4.27	6.00	−1.121	0.262	5.50 ± 4.37	5.00	−2.041	0.041 *
Emotional	9.80 ± 5.14	9	6.73 ± 3.58	8	−2.626	0.009 *	5.43 ± 3.99	3.00	−1.703	0.089	6.83 ± 3.66	6.00	−1.682	0.093
Stigma	3.87 ± 3.25	4	2.33 ± 2.44	2	−1.736	0.083	2.57 ± 2.44	2.00	−1.761	0.078	2.83 ± 2.79	2.50	−1.289	0.197
Social support	1.07 ± 1.62	0	0.67 ± 0.98	0	−1.292	0.196	0.86 ± 1.21	0.00	−0.707	0.480	1.00 ± 1.26	0.50	−0.816	0.414
Cognition	4.40 ± 3.02	4	3.87 ± 2.80	3	−0.984	0.325	5.57 ± 3.10	5.00	−0.541	0.589	5.83 ± 3.82	5.00	−1.089	0.276
Communication	3.13 ± 2.56	2	2.00 ± 1.46	2	−2.288	0.022 *	2.57 ± 1.72	3.00	−1.000	0.317	2.67 ± 2.16	2.50	−0.707	0.480
Bodily discomfort	4.80 ± 2.43	5	2.67 ± 1.76	2	−2.924	0.003 *	2.86 ± 2.04	3.00	−2.264	0.024 *	3.83 ± 1.83	3.00	−1.857	0.063
SF-12														
PCS12	40.43 ± 11.33	40.2	46.74 ± 8.65	48.22	−2.794	0.005 *	46.69 ± 4.93	48.22	−1.859	0.063	46.20 ± 4.22	46.38	−1.782	0.075
MCS12	42.25 ± 8.11	40.33	43.94 ± 8.52	41.7641.76	−0.596	0.551	42.70 ± 9.89	43.57	−0.676	0.499	40.35 ± 8.65	43.63	−0.314	0.753
NPRS														
ACTIVE_right	3.14 ± 3.93	0.00	0.86 ± 1.46	0.00	−1.604	0.109	1.14 ± 2.04	0.00	−1.604	0.109	1.33 ± 2.16	0.00	−1.342	0.180
PASSIVE_right	1.71 ± 2.21	0.00	0.29 ± 0.76	0.00	−1.633	0.102	0.29 ± 0.76	0.00	−1.633	0.102	0.50 ± 1.22	0.00	−1.342	0.180
ACTIVE_left	3.86 ± 2.79	5.00	0.43 ± 1.13	0.00	−2.041	0.041 *	1.14 ± 1.57	0.00	−2.041	0.041 *	1.67 ± 2.07	1.00	−1.841	0.066
PASSIVE_left	1.14 ± 1.57	0.00	0.00 ± 0.00	0.00	−1.633	0.102	0.00 ± 0.00	0.00	−1.633	0.102	0.50 ± 1.22	0.00	−1.633	0.102

* *p* < 0.05. sd = standard deviation.

**Table 2:** Wilcoxon test at T0 and T1 (5 weeks), T2 (9 weeks), and T3 (17 weeks) for range of motion (ROM).

	T0		T1		Wilcoxon T0–T1	*p*	T2		Wilcoxon T0–T2	*p*	T3		Wilcoxon T0–T3	*p*
	Mean ± sd	Median	Mean ± sd	Median			Mean ± sd	Median			Mean ± sd	Median		
PASSIVE RIGHT														
FLEXION	149.58 ± 37.8	160	172.92 ± 11.17	180	−2.214	0.027 *	168.57 ± 15.47	180	−2.041	0.041 *	171.67 ± 14.38	180.00	−1.841	0.066
ABDUCTION	141.67 ± 43.5	155	168.33 ± 19.46	180	−2.207	0.027 *	162.86 ± 23.60	180	−2.032	0.042 *	168.33 ± 20.41	180.00	−1.841	0.066
ADDUCTION	45 ± 0	45	45 ± 0	45	0	10	45 ± 0	45	0	1	45.00 ± 0.00	45.00	0.000	1.000
ROTATION_ASER	53.75 ± 23.6	45	65 ± 18.83	65	−2.816	0.005 *	55.71 ± 12.39	55	−2.388	0.017 *	58.33 ± 11.25	57.50	−2.214	0.027 *
INTERNAL_ROTATION	70 ± 0	70	70 ± 0	70	0	10	70 ± 0	70	0	1	70.00 ± 0.00	70.00	0.000	1.000
EXTENSION	56.67 ± 8.9	60	58.33 ± 5.77	60	−1.414	0.157	60 ± 0	60	0	1	60.00 ± 0.00	60.00	0.000	1.000
ACTIVE RIGHT														
FLEXION	132.08 ± 38.40	127.50	161.25 ± 25.60	172.50	−2.944	0.003 *	156.43 ± 21.55	160	−2.214	0.027 *	155.83 ± 23.33	160.00	−2.032	0.042 *
ABDUCTION	123.75 ± 47.92	105	155.83 ± 32.67	172.50	−2.673	0.008 *	151.43 ± 28.68	160	−2.214	0.027 *	149.17 ± 28.71	155.00	−2.023	0.043 *
ADDUCTION	43.75 ± 4.33	45	44.42 ± 2.02	45	−10	0.317	45 ± 0	45	0	1	45.00 ± 0.00	45.00	0.000	1.000
ROTATION_ASER	45.83 ± 26.70	40	64.58 ± 20.05	67.50	−2.943	0.003 *	55 ± 14.72	60	−2.375	0.018 *	53.33 ± 14.72	55.00	−1.992	0.046 *
INTERNAL_ROTATION	65 ± 10.69	70	70 ± 0	70	−10	0.317	51.43 ± 15.74	60	−1.604	0.317	70.00 ± 0.00	70.00	0.000	1.000
EXTENSION	42.92 ± 19.59	55	55.83 ± 9.96	60	−2.214	0.027 *	156.43 ± 21.55	160	−2.214	0.109	53.33 ± 16.33	60.00	−1.342	0.180
PASSIVE LEFT														
FLEXION	131 ± 44.65	137.50	173 ± 9.49	180	−2.366	0.018 *	172.86 ± 9.51	180	−2.023	0.043 *	175.00 ± 8.37	180.00	−1.826	0.068
ABDUCTION	135 ± 43.46	130	167.50 ± 17.20	180	−2.214	0.027 *	165 ± 19.36	180	−2.041	0.041 *	170.00 ± 15.49	180.00	−1.841	0.066
ADDUCTION	45 ± 0	45	45 ± 0	45	0	10	45 ± 0	45	0	1	45.00 ± 0.00	45.00	0.000	1.000
ROTATION_ASER	52.90 ± 20.81	55	66.20 ± 16.83	70	−2.536	0.011 *	60.71 ± 13.67	70	−2.214	0.027 *	65.00 ± 8.37	70.00	−2.032	0.042 *
INTERNAL_ROTATION	70 ± 0	70	70 ± 0	70	0	10	70 ± 0	70	0	1	70.00 ± 0.00	70.00	−1.000	0.317
EXTENSION	58 ± 6.32	60	58 ± 6.32	60	0	10	60 ± 0	60	0	1	60.00 ± 0.00	60.00	−1.633	0.102
ACTIVE LEFT														
FLEXION	121.50 ± 35.28	117.50	155 ± 26.87	160	−2.677	0.007 *	155.71 ± 20.90	160	−2.375	0.018 *	157.50 ± 19.17	157.50	−2.207	0.027 *
ABDUCTION	110.50 ± 41.33	95	151.50 ± 32.24	162.50	−2.670	0.008 *	152.14 ± 25.63	155	−2.371	0.018 *	156.67 ± 18.89	155.00	−2.207	0.027 *
ADDUCTION	43.50 ± 4.74	45	44.50 ± 1.58	45	−10	0.317	45 ± 0	45	0	1	45.00 ± 0.00	45.00	0.000	1
ROTATION_ASER	40.50 ± 17.39	40	63.50 ± 19.16	65	−2.807	0.003 *	59.29 ± 14.27	60	−2.371	0.018 *	61.67 ± 9.31	62.50	−2.032	0.042 *
INTERNAL_ROTATION	65.71 ± 11.34	70	70 ± 0	70	−10	0.317	70 ± 0	70	−10	0.317	70.00 ± 0.00	70.00	−1.000	0.317
EXTENSION	38.50 ± 23.10	42.50	55 ± 10.80	60	−2.032	0.042 *	57.14 ± 7.56	60	−1.841	0.066	56.67 ± 8.16	60.00	−1.633	0.102

* *p* < 0.05. sd = standard deviation; ASER = arm-at-side external rotation.

**Table 3:** Wilcoxon test at T0 and T1 (5 weeks), T2 (9 weeks), and T3 (17 weeks) for Medical Research Council (MRC) scale.

	T0		T1		Wilcoxon T0–T1	*p*	T2		Wilcoxon T0–T2	*p*	T3		Wilcoxon T0–T3	*p*
	Mean ± sd	Median	Mean ± sd	Median			Mean ± sd	Median			Mean ± sd	Median		
MRC														
RIGHT DELTOID	4.25 ± 0.75	4.00	4.75 ± 0.45	5.00	−2.449	0.014 *	4.29 ± 0.76	4.00	−1.414	0.157	4.33 ± 0.82	4.50	−1.000	0.317
RIGHT BICEPS BRACHII	4.42 ± 0.79	5.00	4.75 ± 0.45	5.00	−2.000	0.046 *	4.43 ± 0.53	4.00	−1.414	0.157	4.50 ± 0.55	4.50	−1.000	0.317
RIGHT ROTATOR CUFF	4.08 ± 0.79	4.00	4.58 ± 0.51	5.00	−2.449	0.014 *	4.14 ± 0.90	4.00	−1.000	0.317	4.33 ± 0.82	4.50	−1.000	0.317
RIGHT TRICEPS	4.33 ± 0.78	4.50	4.58 ± 0.51	5.00	−1.732	0.083	4.71 ± 0.49	5.00	−1.890	0.059	4.67 ± 0.52	5.00	−1.732	0.083
RIGHT TRAPEZIUS	4.25 ± 0.75	4.00	4.58 ± 0.51	5.00	−2.000	0.046 *	4.71 ± 0.49	5.00	−1.890	0.059	4.67 ± 0.52	5.00	−1.732	0.083
RIGHT ANTERIOR SERRATED	4.17 ± 0.83	4.00	4.58 ± 0.51	5.00	−2.236	0.025 *	4.43 ± 0.53	4.00	−1.732	0.083	4.50 ± 0.55	4.50	−1.414	0.157
RIGHT PECTORALIS GRAIN	4.25 ± 0.75	4.00	4.83 ± 0.39	5.00	−2.646	0.008 *	4.33 ± 0.82	4.50	0.000	1.000	4.33 ± 0.52	4.00	0.000	1.000
RIGHT DORSAL	4.50 ± 0.67	5.00	4.83 ± 0.39	5.00	−2.000	0.046 *	4.50 ± 0.55	4.50	−0.577	0.564	4.33 ± 0.52	4.00	0.000	1.000
RIGHT RHOMBHOID	4.18 ± 0.75	4.00	4.58 ± 0.51	5.00	−2.236	0.025 *	4.33 ± 0.82	4.50	−1.000	0.317	4.33 ± 0.52	4.00	−0.577	0.564
LEFT DELTOID	3.90 ± 0.88	4.00	4.60 ± 0.52	5.00	−2.333	0.020 *	4.43 ± 0.53	4.00	−2.121	0.034 *	4.17 ± 0.75	4.00	−2.000	0.046 *
LEFT BICEPS BRACHII	4.30 ± 0.67	4.00	4.80 ± 0.42	5.00	−2.236	0.025 *	4.71 ± 0.49	5.00	−2.000	0.046 *	4.67 ± 0.52	5.00	−1.732	0.083
LEFT ROTATOR CUFF	3.70 ± 0.67	4.00	4.50 ± 0.53	4.50	−2.530	0.011 *	4.29 ± 0.49	4.00	−1.890	0.059	3.83 ± 0.75	4.00	−1.414	0.157
LEFT TRICEPS	4.20 ± 0.63	4.00	4.80 ± 0.42	5.00	−2.449	0.014 *	4.71 ± 0.49	5.00	−2.236	0.025 *	4.67 ± 0.52	5.00	−2.000	0.046 *
LEFT TRAPEZIUS	4.10 ± 0.57	4.00	4.60 ± 0.52	5.00	−2.236	0.025 *	4.43 ± 0.53	4.00	−2.000	0.046 *	4.33 ± 0.52	4.00	−1.732	0.083
LEFT ANTERIOR SERRATED	3.70 ± 0.67	4.00	4.40 ± 0.52	4.00	−2.333	0.020 *	4.29 ± 0.49	4.00	−1.890	0.059	4.00 ± 0.63	4.00	−1.732	0.083
LEFT PECTORALIS GRAIN	4.20 ± 0.63	4.00	4.80 ± 0.42	5.00	−2.449	0.014 *	4.86 ± 0.38	5.00	−2.000	0.046 *	4.83 ± 0.41	5.00	−1.732	0.083
LEFT DORSAL	4.40 ± 0.52	4.00	4.80 ± 0.42	5.00	−2.000	0.046 *	4.86 ± 0.38	5.00	−2.000	0.046 *	4.67 ± 0.52	5.00	−1.414	0.157
LEFT RHOMBHOID	3.67 ± 0.71	4.00	4.60 ± 0.52	5.00	−2.460	0.014 *	4.57 ± 0.53	5.00	−2.271	0.023 *	4.17 ± 0.75	4.00	−1.890	0.059

* *p* < 0.05. sd = standard deviation.

## Data Availability

The data that support the findings of this study are available from the corresponding author upon reasonable request.
